# Adverse Childhood Experience Is Associated With Disrupted White Matter Integrity in Autism Spectrum Disorder: A Diffusion Tensor Imaging Study

**DOI:** 10.3389/fpsyt.2021.823260

**Published:** 2022-01-03

**Authors:** Hiroaki Yoshikawa, Soichiro Kitamura, Kiwamu Matsuoka, Masato Takahashi, Rio Ishida, Naoko Kishimoto, Fumihiko Yasuno, Yuka Yasuda, Ryota Hashimoto, Toshiteru Miyasaka, Kimihiko Kichikawa, Toshifumi Kishimoto, Manabu Makinodan

**Affiliations:** ^1^Department of Psychiatry, School of Medicine, Nara Medical University, Kashihara, Japan; ^2^Department of Functional Brain Imaging, Institute for Quantum Medical Science, National Institutes for Quantum Science and Technology, Chiba, Japan; ^3^Department of Psychiatry, National Center for Geriatrics and Gerontology, Obu, Japan; ^4^Department of Pathology of Mental Diseases, National Institute of Mental Health, National Center of Neurology and Psychiatry, Tokyo, Japan; ^5^Medical Cooperation Foster, Osaka, Japan; ^6^Department of Radiology, Nara Medical University, Kashihara, Japan

**Keywords:** autism spectrum disorder, adverse childhood experiences, white matter, cingulum, uncinate fasciculus, anterior thalamic radiation

## Abstract

Individuals with autism spectrum disorder (ASD) have an increased risk of adverse childhood experiences (ACEs) than typically developed (TD) children. Since multiple lines of studies have suggested that ACEs are related to myelination in the frontal lobe, an exposure to ACEs can be associated with white matter microstructural disruption in the frontal lobe, which may be implicated in subsequential psychological deficits after the adulthood. In this study, we investigated the relationship between ACEs and microstructural integrity on frontal lobe-related white matter tracts using diffusion tensor imaging in 63 individuals with ASD and 38 TD participants. Using a tractography-based analysis, we delineated the uncinate fasciculus (UF), dorsal cingulum (Ci), and anterior thalamic radiation (ATR), which are involved in the neural pathology of ASD, and estimated each diffusion parameter. Compared to the TD participants, individuals with ASD displayed significantly lower fractional anisotropy (FA) and higher radial diffusivity (RD) in the left ATR. Then, ASD individuals exposed to severe ACEs displayed higher RD than those exposed to mild ACEs and TD participants in the left ATR. Moreover, the severity of ACEs, particularly neglect, correlated with lower FA and higher RD in the left UF and ATR in individuals with ASD, which was not observed in TD participants. These results suggest that an exposure to ACEs is associated with abnormality in the frontal lobe-related white matter in ASD.

## Introduction

Autism spectrum disorder (ASD) is a major neurodevelopmental disorder characterized by impaired social communication and restricted repetitive behaviors (1). These characteristics often make it difficult to establish appropriate interpersonal relationships in daily and social life. Individuals with ASD demonstrate atypical structural and functional brain patterns than their typically developed (TD) counterparts and altered network connectivity is involved in the core and other related symptoms of ASD (2). In particular, abnormalities of the neural pathways connected to the frontal lobe are reportedly associated with the pathophysiology of ASD (3–6). Diffusion tensor imaging (DTI) studies have reported on white matter microstructural alteration in the frontal lobe-related white matter bundles in ASD, such as the corpus callosum, uncinate fasciculus (UF), arcuate fasciculus, anterior thalamic radiation (ATR), and cingulum (Ci) (7, 8).

Poor social ability can be associated with an increased risk of exposure to adverse life experiences in ASD. Children with developmental disabilities, including ASD, are likely to experience maltreatment, bullying, and maladaptation in the local community and social life (9). Thus, these adverse childhood experiences (ACEs) reportedly cause poor self-esteem and motivation, thus resulting in subsequent psychiatric comorbidities, such as depression, anxiety, and substance abuse in adults with ASD (10).

Researchers have reported on the relationship between ACE exposure and white matter microstructural disruption. In animal studies, juvenile stress such as social isolation or traumatic stress induced hypomyelination in the prefrontal cortex of mice (11–13). In human studies, an exposure to ACEs influenced white matter microstructural abnormalities in the anterior cingulate cortex, ventromedial prefrontal cortex, corpus callosum, corona radiata, inferior longitudinal fasciculus, and inferior occipitofrontal fasciculus (7, 14–16). While an exposure to neglect in childhood is associated with deteriorated frontal white matter microstructure (6), parental verbal abuse is related to white matter microstructural abnormality of the left arcuate fasciculus (17). Thus, serious maltreatment in early life stages is associated with white matter microstructural abnormalities. However, the mechanism by which an exposure to ACEs influences abnormal white matter microstructure in individuals with ASD has not been completely elucidated.

The relationship between ACEs and white matter microstructural disruption is of clinical importance for considering the pathological basis of ASD. In this study, we compared the association between ACEs and white matter microstructural disruption in individuals with ASD and TD participants. We focused on the UF, Ci, and ATR, which are white matter tracts connected to the frontal lobe. The UF is a hook-shaped bundle of nerves that connects the prefrontal cortex to the medial temporal region (18, 19). It is involved in visual and emotional memory, processing, and decision making (19–21). Regarding the Ci, the dorsal Ci connecting the anterior to the posterior cingulate cortex was explored in this study because it has been involved in emotion and executive control, which was important for ASD characteristics (8, 22). The ATR connects the anterior thalamic nuclei to the prefrontal cortex and is involved in executive function, the planning of complex behaviors, and emotional regulation (23, 24). Previous reports demonstrating the relationship between ACEs and white matter microstructural disruption of these tracts led us to perform this study (8, 25). We hypothesized that individuals with ASD exposed to serious ACEs display more severe white matter microstructural disruption than those exposed to mild ACEs and TD participants.

## Methods

### Participants

We enrolled 63 age- and intelligence quotient (IQ)-matched individuals with ASD and 38 TD participants. The full-scale IQ of the participants was estimated using similarities and symbol search subsets of the Wechsler Adult Intelligence Scale, third edition (26). To match the IQ between individuals with ASD and TD participants, those with IQ <80 and >120 were excluded from the study. Individuals with ASD were recruited from the outpatient service of the Department of Psychiatry, Nara Medical University Hospital and affiliated psychiatric clinics in Japan. They were diagnosed by two trained psychiatrists based on the criteria of the Diagnostic and Statistical Manual-5 and the Japanese version of the Autism Diagnostic Observation Schedule, second edition (27), and autistic traits were also examined by the Autism-Spectrum Quotient Japanese version (AQ-J) (28). Twenty individuals with ASD had neuropsychiatric comorbidities, including major depressive disorder (*n* = 6), attention deficit hyperactivity disorder (*n* = 4), adjustment disorder (*n* = 4), anxiety disorder (*n* = 3), avoidant personality disorder (*n* = 2), alcohol use disorder (*n* = 1), epilepsy (*n* = 1), schizophrenia (*n* = 1), bipolar disorder (*n* = 1), panic disorder (*n* = 1), obsessive compulsive disorder (*n* = 1), oppositional defiant disorder (*n* = 1), and learning deficits (*n* = 1). TD participants were recruited from the public offering of the students, hospital, and school staff of the Nara Medical University. They did not have a history of psychiatric, neurological, or developmental disorders, and were requested to complete the Mini-International Neuropsychiatric Interview to exclude their current or past psychiatric history. Moreover, we evaluated them using the AQ-J, and a score <32 was used as the enrollment requirement. Thirty-four individuals with ASD were prescribed the following psychotropic medications during their participation: antidepressants (*n* = 21), hypnotic agents (*n* = 16), antipsychotics (*n* = 13), anti-anxiety agents (*n* = 12), anti-epileptic agents (*n* = 3), atomoxetine (*n* = 2), and anti-manic agents (*n* = 1). None of the TD participants had a history of psychotropic medications. Structural abnormalities of the brain were excluded in both groups, as determined by T1-weighted magnetic resonance imaging (MRI). We assessed the severity of ACEs using the Japanese version of the Child Abuse and Trauma Scale (CATS) (29). The CATS is a 38-item instrument that retrospectively evaluates adverse childhood experiences (30). Each item is measured on a five-point scale ranging from 0 to 4 and is divided into five major factors of adverse childhood experiences as follows: neglect or negative home atmosphere, sexual abuse, punishment, emotional abuse, and others. In addition, the sum of the scores provides the total score. There was no cut-off point on the scale, and we measured the median of the total CATS score in individuals with ASD. Considering the median score was 37, individuals with ASD and total CATS score ≥38 were defined as experiencing higher ACEs (ASD with high CATS). In contrast, those with scores ≤37 were defined as experiencing lower ACEs (ASD with low CATS) to explore the relationship between the severity of ACEs and microstructural white matter alteration. This study was approved by the Institutional Review Board of Nara Medical University, and all analyses were performed in accordance with relevant guidelines and regulations. Written informed consent was obtained from all individuals prior to their participation in the study.

### MRI Data Acquisition

All participants underwent brain MRI using a 3-Tesla clinical scanner equipped with a 32 phased-array head coil (Magnetom Verio; Siemens, Erlangen, Germany). The participants were scanned with a three-dimensional T1-weighted gradient echo sequence (repetition time [TR] = 1,900 ms; echo time [TE] = 2.54 ms; field of view [FOV] = 256 × 256 mm; acquisition matrix = 256 × 256; and 208 contiguous axial slices of 1 mm thickness). We acquired DT images with an echo-planar imaging sequence using a GeneRalized Autocalibrating Partially Parallel Acquisition factor of two. The imaging parameters were as follows: TR = 14,100 ms, TE = 81 ms, FOV = 256 × 256 mm, acquisition matrix = 128 × 128, 79 contiguous axial slices of 2 mm thickness, b = 1,000 s/mm^2^, and 30-axis encoding.

### Image Processing

**Figure 1:**
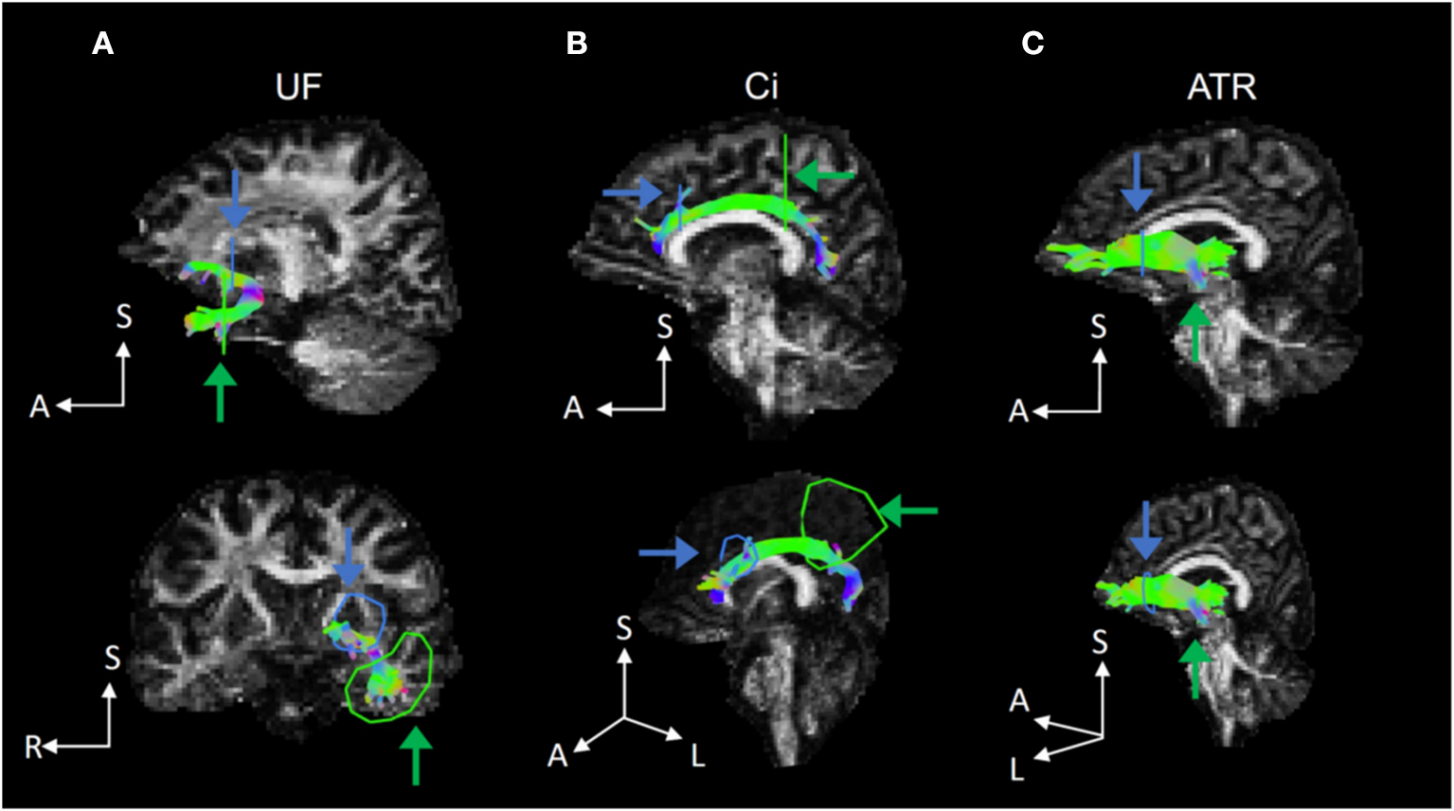
Representative fiber tracts delineated by deterministic tractography for the UF, Ci, and ATR. **(A)** In the UF, we set a seed region of interest (ROI) in the white matter on a coronal plane at the tip of the inferior horn of the lateral ventricle and a target ROI in the white matter on a coronal plane at the tip of the frontal horn of the lateral ventricle. **(B)** In the Ci, we set a seed ROI in the white matter on a coronal plane on the top of the genu of the corpus callosum and a target ROI in the white matter on a coronal plane on the top of the splenium of the corpus callosum. **(C)** In the ATR, we set a seed ROI in the white matter on a coronal plane at the anterior limb of internal capsule and a target ROI in the white matter on a coronal plane at the anterior edge of pons. UF, uncinate fasciculus; Ci, cingulum; ATR, anterior thalamic radiation.

DTI was performed using the ExploreDTI software (https://www.exploredti.com/). It included corrections for head motion and eddy current-induced geometric distortions of raw diffusion-weighted data (31). We estimated the diffusion tensor using a non-linear least squares approach (32). We delineated the UF, Ci, and ATR using deterministic tractography. In tractography, the region of interest was set according to a previous study (Figure 1) (33). In each of the delineated fiber tracts, we calculated three diffusion parameters as follows: fractional anisotropy (FA), mean diffusivity (MD), and radial diffusivity (RD). To assess the head motion, we evaluated the root mean square (RMS) deviation of absolute intervolume displacement with respect to the b = 0 images from intra-participant registration parameters using the rmsdiff tool in FSL (34). The average displacement distance between each consecutive pair of 31 volumes was calculated for each participant.

### Statistical Analyses

For continuous variables in the demographic data, group comparisons were performed using unpaired two-tailed *t*-tests and Mann–Whitney U tests for normally distributed and non-normally distributed data, respectively. For categorical variables, we performed Fisher's exact test to compare the groups. Group differences in the estimated diffusion parameters in the UF, Ci, and ATR were assessed using the analysis of covariance (ANCOVA) for years of education and the AQ-J as covariates between individuals with ASD and TD participants with Bonferroni correction to avoid type I errors due to multiplicity (*p* < 0.0083). Additionally, regarding the group differences for the comparison of ASD with low and high CATS score and TD participants, the estimated diffusion parameters in the UF, Ci, and ATR were assessed using the analysis of covariance (ANCOVA) for years of education and the AQ-J as covariates with Bonferroni correction to avoid type I errors due to multiplicity (*p* < 0.0083), additionally with post *hoc* Fisher's least significant difference test (*p* < 0.05) for exploring each of the group difference. To explore the relationships between the severity of ACEs and the extent of white matter microstructural alteration, we performed partial Spearman's rank correlation analyses. This helped us examine the relationships between CATS scores and estimated diffusion parameters in the UF, Ci, and ATR with years of education and the AQ-J as covariates in individuals with ASD and TD participants, respectively (*p* < 0.05, as significant). Normality assumptions for the statistical analysis were evaluated using the Shapiro–Wilk test for each dataset. The analyses were performed using SPSS version 27 (IBM Inc., Armonk, NY, USA).

## Results

### Demographic Characteristics

Table 1 summarizes the demographic and clinical characteristics of the study participants. There were no significant differences in the age, IQ, sex, and handedness between the two groups (*p* > 0.05). In contrast, individuals with ASD displayed significantly lesser years of education and higher AQ-J scores than TD participants (*p* < 0.05). Individuals with ASD demonstrated significantly higher scores on each of the CATS scales than TD participants (*p* < 0.05). There were no significant differences in the age, IQ, years of education, sex, handedness, and AQ-J and ADOS-2 scores between those with ASD with high and low CATS scores (Supplementary Table 1). There was no significant difference in the extent of head motion based on the average RMS distance between individuals with ASD and TD participants (ASD: 1.4 ± 0.2 mm, TD: 1.4 ± 0.3 mm, U = 1,072, *p* = 0.38). Additionally, no significant difference of average RMS distance was shown between ASD individuals with low and high ASD (ASD individuals with low CATS: 1.4 ± 0.2 mm, TD: 1.4 ± 0.2 mm, U = 492, *p* = 0.96).

**Table 1:** Demographic characteristics of the study participants.

	**ASD (*n* = 63)**	**TD (*n* = 38)**	**T or U or χ^2^**	***p*-value**
Age (mean, SD)	27.3 (5.6)	27.8 (5.6)	1342.5	0.31
Duration of education (mean, SD)	15.0 (2.3)	16.2 (2.3)	1535	0.015^*^
IQ (mean, SD)	101.0 (12.0)	104.8 (9.6)	1438.5	0.09
Sex (male, %)	48 (76.2)	27 (71.1)	0.33	0.64
Handedness (right, %)	58 (92.1)	38 (100)	3.2	0.15
AQ-J (mean, SD)	31.4 (7.4)	18.4 (7.0)	253	<0.001^*^
ADOS-2 (mean, SD)	15.4 (3.1)	N/A	N/A	N/A
CATS (mean, SD)				
Total	44.0 (25.7)	20.8 (14.4)	455	<0.001^*^
Punishment	10.1 (5.1)	7.8 (3.6)	860	0.018^*^
Sexual abuse	0.56 (1.3)	0.08 (0.4)	989	0.021^*^
Neglect	15.2 (9.7)	6.9 (6.8)	545	<0.001^*^
Emotional abuse	11.0 (7.8)	3.8 (3.1)	459	<0.001^*^
Others	7.1 (6.0)	2.2 (3.4)	463	<0.001^*^

*ASD, autism spectrum disorder; TD, typically developed; IQ, Intelligence quotient; AQ-J, Autism-Spectrum Quotient - Japanese version; ADOS-2, Autism Diagnostic Observation Schedule second edition; CATS, Child Abuse and Trauma Scale; SD, standard deviation*.

* *p < 0.083*.

### Group Comparisons of Each DTI Parameter of the Three Tracts Between Individuals With ASD and TD Participants

Table 2 outlines group comparisons of DTI parameters between individuals with ASD and TD participants. The ANCOVA revealed that the diagnosis exerted a statistically significant effect, such that individuals with ASD revealed lower FA (*F*[2, 97] = 21.0; *p* < 0.001) and higher RD (*F*[2, 97] = 9.0; *p* = 0.003) in the left ATR than TD participants. There were no significant differences in the DTI parameters in the UF and Ci between the groups (*p* > 0.0083).

**Table 2:** Group comparisons of each diffusion parameter in individuals with ASD and TD participants.

		**ASD**	**TD**	***F***	***p*-value**
UF	Right FA	0.410 (0.019)	0.420 (0.022)	2.2	0.14
	Left FA	0.409 (0.020)	0.417 (0.019)	0.64	0.43
	Right MD	0.720 (0.028)	0.725 (0.027)	0.27	0.61
	Left MD	0.744 (0.026)	0.752 (0.023)	0	0.99
	Right RD	0.547 (0.024)	0.546 (0.027)	1.1	0.29
	Left RD	0.565 (0.023)	0.566 (0.023)	0.14	0.71
Ci	Right FA	0.511 (0.035)	0.531 (0.029)	0.62	0.43
	Left FA	0.458 (0.043)	0.483 (0.036)	5.1	0.026
	Right MD	0.685 (0.031)	0.698 (0.022)	0.13	0.72
	Left MD	0.675 (0.030)	0.683 (0.026)	0.95	0.33
	Right RD	0.467 (0.031)	0.463 (0.029)	0.079	0.78
	Left RD	0.489 (0.029)	0.483 (0.030)	4.9	0.03
ATR	Right FA	0.417 (0.025)	0.423 (0.020)	0.046	0.83
	Left FA	0.418 (0.018)	0.441 (0.020)	21.0	<0.001^*^
	Right MD	0.688 (0.023)	0.689 (0.024)	1.2	0.27
	Left MD	0.688 (0.031)	0.689 (0.024)	0.13	0.72
	Right RD	0.521 (0.023)	0.519 (0.023)	0.75	0.39
	Left RD	0.521 (0.019)	0.514 (0.021)	9.0	0.003^*^

*ASD, autism spectrum disorder; TD, typically developed; UF, uncinate fasciculus; Ci, Cingulum; ATR, anterior thalamic radiation; FA, fractional anisotropy; MD, mean diffusivity; RD, radial diffusivity*.

* *p < 0.05*.

### Group Comparisons of Each DTI Parameter of the Three Tracts Among Individuals With ASD With High and Low CATS Scores and TD Participants

**Figure 2:**
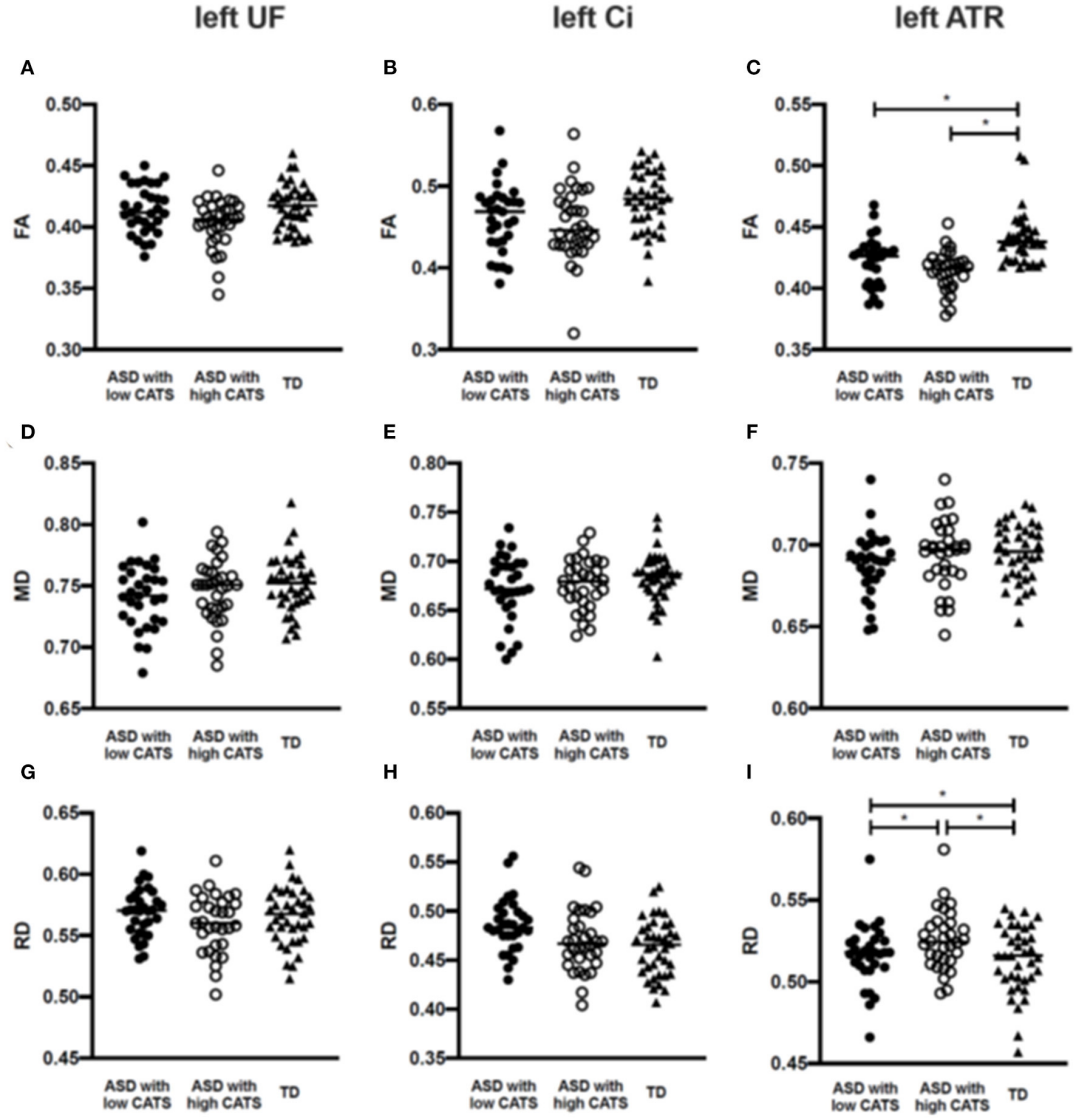
Group comparisons of the estimated diffusion parameters among individuals with ASD with low and high CATS scores and TD participants. **(A–C)** Individuals with ASD with low and high CATS scores display significantly lower FA than TD participants. No significant differences are shown between individuals with ASD with low and high CATS scores. **(D–F)** There are no significant differences of MD among individuals with ASD and TD participants. **(G–I)** Individuals with ASD with low and high CATS scores display significantly higher RD than TD participants. Those with ASD and high CATS scores also display higher RD than those with low CATS scores. *p<0.05. ASD, autism spectrum disorder; TD, typically developed; CATS, Child Abuse and Trauma Scale; FA, fractional anisotropy; RD, radial diffusivity; ATR, anterior thalamic radiation.

Figure 2 and Supplementary Table 2 summarize the group comparisons of each DTI parameter among individuals with ASD having high and low CATS scores and TD participants. The ANCOVA revealed that the diagnosis exerted a statistically significant effect, such that individuals with ASD revealed lower FA (*p* < 0.001) and higher RD in the left ATR (*p* = 0.002). *Post hoc* comparisons revealed that those with ASD with high (*p* < 0.001) and low (*p* < 0.001) CATS scores demonstrated significantly lower FA in the left ATR than TD participants. In contrast, there was no significant difference in FA in the left ATR between individuals with ASD with high and low CATS scores (*p* = 0.12). Moreover, those with ASD and high CATS scores demonstrated significantly higher RD in the left ATR than those with low CATS scores (*p* = 0.04) and TD participants (*p* < 0.001). In addition, individuals with ASD and low CATS scores demonstrated significantly higher RD than TD participants (*p* = 0.044). There were no significant differences in MD in the left ATR among the groups (*p* > 0.05). Furthermore, we did not observe significant differences in the diffusion parameters in the right ATR (*p* > 0.05). Then, there were no significant diagnostic effect on the UF and Ci among the three groups (*p* > 0.0083).

### Significant Association Between the Severity of CATS Scores in ASD and Diffusion Parameters in the UF and ATR

**Figure 3:**
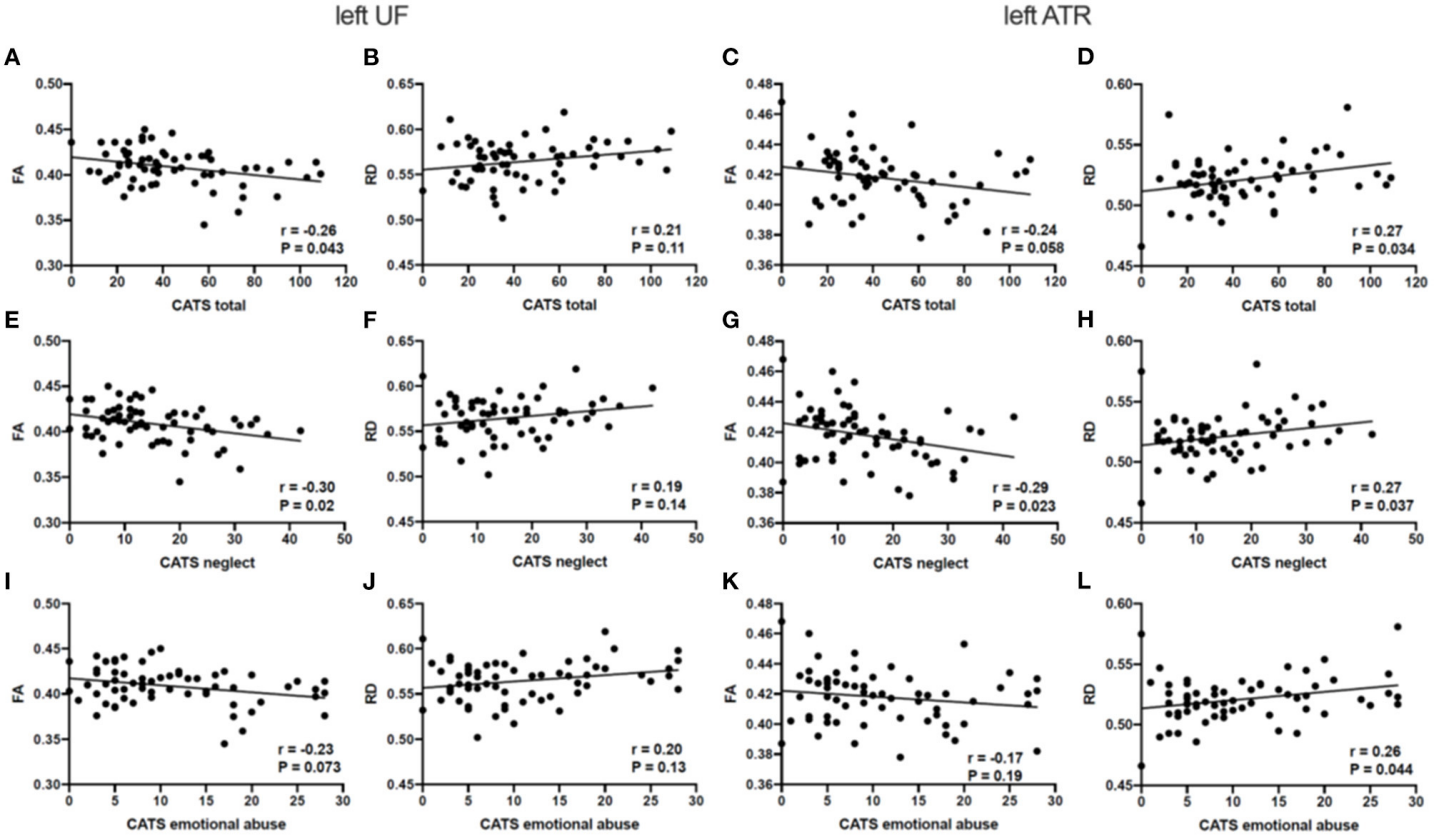
Relationships between the estimated diffusion parameters and each CATS score in ASD. **(A–D)** RD in the left ATR is significantly correlated with the total CATS score in ASD. **(E–H)** FA in the left UF and ATR, and RD in the left ATR display significant correlations with the neglect CATS subscale scores in ASD. **(I–L)** RD in the left ATR is significantly correlated with the emotional abuse CATS subscale scores in ASD. *p* < 0.05 as statistically significant. CATS, Child Abuse and Trauma Scale; FA, fractional anisotropy; RD, radial diffusivity; UF, uncinate fasciculus; ATR. anterior thalamic radiation.

Figure 3 outline Spearman's partial correlation analyses between the CATS total scores and subscale scores in individuals with ASD and TD participants. The CATS total score was negatively correlated with FA in the left UF (*p* = 0.043) and positively correlated with RD in the left ATR (*p* = 0.034) in individuals with ASD. There were no significant correlations between the CATS total score and each diffusion parameter in the UF and ATR in TD participants (*p* > 0.05). While the neglect subscale score was negatively correlated with FA in the left UF (*p* = 0.02) and ATR (*p* = 0.023), it was positively correlated with RD in the left ATR (*p* = 0.037) in individuals with ASD. Moreover, the emotional abuse subscale score was positively correlated with RD in the left ATR in individuals with ASD (*p* = 0.044). There were no significant correlations between each of the CATS subscale scores and each diffusion parameter in the UF and ATR in TD participants (*p* > 0.05).

## Discussion

In the present study, individuals with ASD demonstrated significantly lower FA and higher RD in the left ATR than TD participants, consistent with the findings of previous studies (8, 35, 36). In consideration of ACEs, those with ASD and high CATS scores revealed significantly lower FA and higher RD in the left ATR than TD participants. Moreover, they demonstrated significantly higher RD in the left ATR than those with low CATS scores. ACE severity was correlated with white matter microstructural alterations in the left UF and ATR, of which neglect and demonstrated a significant correlation with the left UF and ATR, in addition emotional abuse indicated a significant correlation with the left ATR. In other words, neglect and emotional abuse were clinically important for white matter development in ASD.

In the DTI parameter, FA reflects less restricted water diffusion of the white matter tract, and reduced FA is associated with disrupted fiber tracts (37). RD reportedly reflects diffusivity perpendicular to axonal fibers, and increased RD is associated with the disruption of the myelin sheath (38–40). Furthermore, decreased FA and increased RD can be considered to reflect the disruption of compacted myelin sheath structure of neural fibers (38, 41). Our findings suggested that individuals with ASD displayed pronounced disruption of compacted myeline sheath in the frontal-related fiber tracts than TD participants, consistent with the findings of previous reports (42). Moreover, ACEs were related to the severity of disrupted myeline sheath in individuals with ASD but not in TD participants. A previous animal study reported that Fmr1 knockout mice, as a possible model of ASD, exhibited excessive sensitivity to environmental changes and synaptic connectivity (43). Therefore, individuals with ASD may be more vulnerable to ACE exposure, in relation to white matter deficits than TD participants.

Individuals with ASD exposed to both severe and mild ACE demonstrated more white matter microstructural disruption than TD participants in the left ATR. In other words, those with ASD fundamentally demonstrated white matter disruption regardless of exposure to ACEs. Moreover, individuals with ASD and exposed to severe ACEs demonstrated worse white matter microstructural abnormality than those exposed to mild ACEs in the left ATR. Therefore, the ATR may be susceptible to ACE exposure, and its effect can be associated with white matter microstructural disruption in ASD. Individuals with ASD and exposed to severe ACEs may be at an increased risk of psychiatric comorbidities, such as depression, anxiety disorder, and posttraumatic stress disorder (3, 10, 44). The ATR dysconnectivity, likely representing cortico-thalamic network dysfunction, can associate with cognitive dysfunction and emotional dysregulation, thereby resulting in psychological symptoms in adults with ASD.

An exposure to neglect in early life stages is associated with white matter abnormality in the prefrontal region (8), and interestingly, it is of note that mouse models of early life neglect show hypomyelination in the prefrontal cortex (11, 12). Our findings demonstrated significant correlations between the severity of ACEs, particularly neglect, and deteriorated DTI parameters in the left UF and ATR. Similar to the ATR, the UF is reportedly associated with psychiatric disorders (6, 45). An exposure to neglect in childhood has been associated with psychiatric comorbidities in adulthood. Then, previous reports demonstrated that an exposure to emotional abuse was also involved in white matter abnormality (46, 47), which were consistent with our results. Our findings suggested that the aforementioned exposure was associated with white matter microstructural abnormalities in the UF and ATR, which may be involved in emotional dysregulation and irregular decision-making and the subsequent appearance of psychological symptoms in ASD. Nonetheless, there were no significant correlations between the severity of ACEs and each DTI parameter in TD participants. Therefore, the susceptibility to ACEs differs between individuals with ASD and their TD counterparts. Moreover, individuals with ASD may present with affected frontal lobe-related white matter on exposure to ACEs.

Our samples demonstrated the laterality of abnormal white matter microstructure and its association with the severity of ACEs in ASD. This laterality has been previously reported in studies on ASD and was consistent with our results (16). A previous study reported that white matter microstructures were dominantly impaired on the left side in the UF and ATR in individuals with ASD exposed to ACEs (42).

This study had several limitations. First, since our work was a cross-sectional study, causal relationships between an exposure to ACEs and white matter microstructural abnormality have not been fully elucidated. Second, the CATS is a self-assessment questionnaire for adverse life events; thus, it might have introduced recall bias in the participants, thereby influencing our findings. Considering the CATS was validated in a previous study, we compared the severity of ACEs among our samples (48). Third, we could not deny the possibility that abnormalities in networks other than the UF, Ci, and ATR were related to ACE exposure. Additionally, some participants with ASD in this study had psychiatric comorbidities, which were related to white matter abnormalities. Future studies are warranted to address these issues.

In conclusion, an exposure to ACEs is more likely to be associated with white matter microstructural disruption in the frontal lobe-related white matter tracts in individuals with ASD than TD participants. Of the ACE types, neglect can be of critical importance for white matter disruption in ASD. Our findings suggested the importance of a comprehensive growth environment based on the consideration of ASD characteristics, which may assist in appropriate neuronal development in ASD patients.

## Data Availability Statement

The raw data supporting the conclusions of this article will be made available by the authors, without undue reservation.

## Ethics Statement

The studies involving human participants were reviewed and approved by the Institutional Review Board of Nara Medical University. The patients/participants provided their written informed consent to participate in this study.

## Author Contributions

HY, SK, and MM wrote the manuscript and undertook the analysis and interpretation of imaging data. MM contributed to the conception, design of this research, and interpretation of the results. KM undertook the analysis and contributed to the interpretation of the results. MT, FY, and TK contributed to the interpretation of the results. TM and KK contributed to the acquisition of MRI data. RI and NK performed psychological evaluation. YY and RH supervised the ADOS-2. All authors contributed to the article and approved the submitted version.

## Funding

This work was supported by the Japanese Society for the Promotion of Science KAKENHI (Grant Numbers: 19K17116 to SK; 16H06403, 16H06400, 16H02666, and 16H05377 to MM), PRIME, AMED under Grant Number JP21gm6310015, AMED under Grant Number 21wm04250XXs0101, and AMED under Grant Number 21uk1024002s0201.

## Conflict of Interest

The authors declare that the research was conducted in the absence of any commercial or financial relationships that could be construed as a potential conflict of interest.

## Publisher's Note

All claims expressed in this article are solely those of the authors and do not necessarily represent those of their affiliated organizations, or those of the publisher, the editors and the reviewers. Any product that may be evaluated in this article, or claim that may be made by its manufacturer, is not guaranteed or endorsed by the publisher.

## References

[1] American Psychiatric Association. Diagnostic and Statistical Manual of Mental Disorders. 5th ed. Arlington: VA: American Psychiatric Association (2013). 10.1176/appi.books.9780890425596

[2] Groen WB Buitelaar JK van der Gaag RJ Zwiers MP. Pervasive microstructural abnormalities in autism: a DTI study. J Psychiatry Neurosci. (2011) 36:32–40. 10.1503/jpn.090100 20964953 PMC3004973

[3] Carr CP Martins CM Stingel AM Lemgruber VB Juruena MF. The role of early life stress in adult psychiatric disorders: a systematic review according to childhood trauma subtypes. J Nerv Ment Dis. (2013) 201:1007–20. 10.1097/NMD.0000000000000049 24284634

[4] Kubo KI. Increased densities of white matter neurons as a cross-disease feature of neuropsychiatric disorders. Psychiatry Clin Neurosci. (2020) 74:166–75. 10.1111/pcn.12962 31788900

[5] Lim L Hart H Howells H Mehta MA Simmons A Mirza K. Altered white matter connectivity in young people exposed to childhood abuse: a tract-based spatial statistics (TBSS) and tractography study. J Psychiatry Neurosci. (2019) 44:E11–E20. 10.1503/jpn.170241 30964614 PMC6606424

[6] Lim L Howells H Radua J Rubia K. Aberrant structural connectivity in childhood maltreatment: a meta-analysis. Neurosci Biobehav Rev. (2020) 116:406–14. 10.1016/j.neubiorev.2020.07.004 32659288

[7] Choi J Jeong B Polcari A Rohan ML Teicher MH. Reduced fractional anisotropy in the visual limbic pathway of young adults witnessing domestic violence in childhood. Neuroimage. (2012) 59:1071–9. 10.1016/j.neuroimage.2011.09.033 21985907 PMC3236680

[8] Tendolkar I Mårtensson J Kühn S Klumpers F Fernández G. Physical neglect during childhood alters white matter connectivity in healthy young males. Hum Brain Mapp. (2018) 39:1283–90. 10.1002/hbm.23916 29250891 PMC6866381

[9] Sullivan PM Knutson JF. Maltreatment and disabilities: a population-based epidemiological study. Child Abuse Negl. (2000) 24:1257–73. 10.1016/S0145-2134(00)00190-3 11075694

[10] Brenner J Pan Z Mazefsky C Smith KA Gabriels R Autism and Developmental Disorders Inpatient Research Collaborative (ADDIRC). Behavioral symptoms of reported abuse in children and adolescents with autism spectrum disorder in inpatient settings. J Autism Dev Disord. (2018) 48:3727–35. 10.1007/s10803-017-3183-4 28593599

[11] Makinodan M Rosen KM Ito S Corfas G. A critical period for social experience-dependent oligodendrocyte maturation and myelination. Science. (2012) 337:1357–60. 10.1126/science.1220845 22984073 PMC4165613

[12] Liu J Dietz K DeLoyht JM Pedre X Kelkar D Kaur J. Impaired adult myelination in the prefrontal cortex of socially isolated mice. Nat Neurosci. (2012) 15:1621–3. 10.1038/nn.3263 23143512 PMC3729624

[13] Breton JM Barraza M Hu KY Frias SJ Long KLP Kaufer D. Juvenile exposure to acute traumatic stress leads to long-lasting alterations in grey matter myelination in adult female but not male rats. Neurobiol Stress. (2021) 14:100319. 10.1016/j.ynstr.2021.100319 33937444 PMC8079662

[14] Tanti A Kim JJ Wakid M Davoli MA Turecki G Mechawar N. Child abuse associates with an imbalance of oligodendrocyte-lineage cells in ventromedial prefrontal white matter. Mol Psychiatry. (2018) 23:2018–28. 10.1038/mp.2017.231 29158585

[15] Tomoda A Navalta CP Polcari A Sadato N Teicher MH. Childhood sexual abuse is associated with reduced gray matter volume in visual cortex of young women. Biol Psychiatry. (2009) 66:642–8. 10.1016/j.biopsych.2009.04.021 19560122 PMC4277202

[16] Lutz PE Tanti A Gasecka A Barnett-Burns S Kim JJ Zhou Y. Association of a history of child abuse with impaired myelination in the anterior cingulate cortex: convergent epigenetic, transcriptional, and morphological evidence. Am J Psychiatry. (2017) 174:1185–94. 10.1176/appi.ajp.2017.16111286 28750583

[17] Samson AC Dougherty RF Lee IA Phillips JM Gross JJ Hardan AY. White matter structure in the uncinate fasciculus: Implications for socio-affective deficits in autism spectrum disorder. Psychiatry Res Neuroimaging. (2016) 255:66–74. 10.1016/j.pscychresns.2016.08.004 27552717

[18] Ghashghaei HT Hilgetag CC Barbas H. Sequence of information processing for emotions based on the anatomic dialogue between prefrontal cortex and amygdala. Neuroimage. (2007) 34:905–23. 10.1016/j.neuroimage.2006.09.046 17126037 PMC2045074

[19] Von Der Heide RJ Skipper LM Klobusicky E Olson IR. Dissecting the uncinate fasciculus: Disorders, controversies and a hypothesis. Brain. (2013) 136:1692–707. 10.1093/brain/awt094 23649697 PMC3673595

[20] Schmahmann JD Weilburg JB Sherman JC. The neuropsychiatry of the cerebellum - Insights from the clinic. Cerebellum. (2007) 6:254–67. 10.1080/14734220701490995 17786822

[21] Koshiyama D Fukunaga M Okada N Morita K Nemoto K Usui K. White matter microstructural alterations across four major psychiatric disorders: mega-analysis study in 2937 individuals. Mol Psychiatry. (2020) 25:883–95. 10.1038/s41380-019-0553-7 31780770 PMC7156346

[22] Metzler-Baddeley C Jones DK Steventon J Westacott L Aggleton JP O'Sullivan MJ. Cingulum microstructure predicts cognitive control in older age and mild cognitive impairment. J Neurosci. (2012) 32:17612–9. 10.1523/JNEUROSCI.3299-12.2012 23223284 PMC6621654

[23] Coenen VA Panksepp J Hurwitz TA Urbach H Mädler B. Human medial forebrain bundle (MFB) and anterior thalamic radiation (ATR): Imaging of two major subcortical pathways and the dynamic balance of opposite affects in understanding depression. J Neuropsychiatry Clin Neurosci. (2012) 24:223–36. 10.1176/appi.neuropsych.11080180 22772671

[24] Floresco SB Grace AA. Gating of hippocampal-evoked activity in prefrontal cortical neurons by inputs from the mediodorsal thalamus and ventral tegmental area. J Neurosci. (2003) 23:3930–43. 10.1523/JNEUROSCI.23-09-03930.2003 12736363 PMC6742171

[25] Bubb EJ Metzler-Baddeley C Aggleton JP. The cingulum bundle: anatomy, function, and dysfunction. Neurosci Biobehav Rev. (2018) 92:104–27. 10.1016/j.neubiorev.2018.05.008 29753752 PMC6090091

[26] Sumiyoshi C Fujino H Sumiyoshi T Yasuda Y Yamamori H Ohi K. Usefulness of the Wechsler intelligence scale short form for assessing functional outcomes in patients with schizophrenia. Psychiatry Res. (2016) 245:371–8. 10.1016/j.psychres.2016.08.018 27591412

[27] Kurita H Koyama T Osada H. Autism-Spectrum Quotient-Japanese version and its short forms for screening normally intelligent persons with pervasive developmental disorders. Psychiatry Clin Neurosci. (2005) 59:490–6. 10.1111/j.1440-1819.2005.01403.x 16048456

[28] Baron-Cohen S Wheelwright S Skinner R Martin J Clubley E. The Autism-Spectrum Quotient (AQ): evidence from asperger syndrome/high-functioning autism, males and females, scientists and mathematicians. J Autism Dev Disord. (2001) 31:5–17. 10.1023/A:1005653411471 11439754

[29] Sanders B Becker-Lausen E. The measurement of psychological maltreatment: Early data on the child abuse and trauma scale. Child Abuse Negl. (1995) 19:315–23. 10.1016/S0145-2134(94)00131-6 9278731

[30] Tanabe H Ozawa S Goto K. Psychometric properties of the Japanese version of the Child Abuse and Trauma Scale (CATS). In: The 9th Annual Meeting of the Japanese Society for Traumatic Stress Studies (in Japanese). Kobe (2010).

[31] Leemans A Jones DK. The B-matrix must be rotated when correcting for subject motion in DTI data. Magn Reson Med. (2009) 61:1336–49. 10.1002/mrm.21890 19319973

[32] Jones DK Basser PJ. ‘Squashing peanuts and smashing pumpkins’: How noise distorts diffusion-weighted MR data. Magn Reson Med. (2004) 52:979–93. 10.1002/mrm.20283 15508154

[33] Wakana S Caprihan A Panzenboeck MM Fallon JH Perry M Gollub RL. Reproducibility of quantitative tractography methods applied to cerebral white matter. Neuroimage. (2007) 36:630–44. 10.1016/j.neuroimage.2007.02.049 17481925 PMC2350213

[34] Jenkinson M Bannister P Brady M Smith S. Improved optimization for the robust and accurate linear registration and motion correction of brain images. Neuroimage. (2002) 17:825–41. 10.1016/S1053-8119(02)91132-8 12377157

[35] Benedetti F Bollettini I Radaelli D Poletti S Locatelli C Falini A. Adverse childhood experiences influence white matter microstructure in patients with bipolar disorder. Psychol Med. (2014) 44:3069–82. 10.1017/S0033291714000506 25065766

[36] Li Y Zhou Z Chang C Qian L Li C Xiao T. Anomalies in uncinate fasciculus development and social defects in preschoolers with autism spectrum disorder. BMC Psychiatry. (2019) 19:399. 10.1186/s12888-019-2391-1 31842898 PMC6916076

[37] Galantucci S Tartaglia MC Wilson SM Henry ML Filippi M Agosta F. White matter damage in primary progressive aphasias: a diffusion tensor tractography study. Brain. (2011) 134:3011–29. 10.1093/brain/awr099 21666264 PMC3187537

[38] Beaulieu C. The basis of anisotropic water diffusion in the nervous system - a technical review. NMR Biomed. (2002) 15:435–55. 10.1002/nbm.782 12489094

[39] Heckel A Weiler M Xia A Ruetters M Pham M Bendszus M. Peripheral nerve diffusion tensor imaging: Assessment of axon and myelin sheath integrity. PLoS ONE. (2015) 10:e0130833. 10.1371/journal.pone.0130833 26114630 PMC4482724

[40] Song SK Sun SW Ramsbottom MJ Chang C Russell J Cross AH. Dysmyelination revealed through MRI as increased radial (but unchanged axial) diffusion of water. Neuroimage. (2002) 17:1429–36. 10.1006/nimg.2002.1267 12414282

[41] Scheel M Prokscha T Bayerl M Gallinat J Montag C. Myelination deficits in schizophrenia: Evidence from diffusion tensor imaging. Brain Struct Funct. (2013) 218:151–6. 10.1007/s00429-012-0389-2 22327232

[42] Niida R Yamagata B Niida A Uechi A Matsuda H Mimura M. Aberrant anterior thalamic radiation structure in bipolar disorder: a diffusion tensor tractography study. Front Psychiatry. (2018) 9:522. 10.3389/fpsyt.2018.00522 30405460 PMC6207644

[43] Dölen G Osterweil E Rao BS Smith GB Auerbach BD Chattarji S. Correction of fragile X syndrome in mice. Neuron. (2007) 56:955–62. 10.1016/j.neuron.2007.12.001 18093519 PMC2199268

[44] Koch SBJ van Zuiden M Nawijn L Frijling JL Veltman DJ Olff M. Decreased uncinate fasciculus tract integrity in male and female patients with PTSD: a diffusion tensor imaging study. J Psychiatry Neurosci. (2017) 42:331–42. 10.1503/jpn.160129 28452713 PMC5573575

[45] Johnson CP Juranek J Kramer LA Prasad MR Swank PR Ewing-Cobbs L. Predicting behavioral deficits in pediatric traumatic brain injury through uncinate fasciculus integrity. J Int Neuropsychol Soc. (2011) 17:663–73. 10.1017/S1355617711000464 21492497 PMC3707392

[46] Eluvathingal TJ Chugani HT Behen ME Juhász C Muzik O Maqbool M. Abnormal brain connectivity in children after early severe socioemotional deprivation: A diffusion tensor imaging study. Pediatrics. (2006) 117:2093–100. 10.1542/peds.2005-1727 16740852

[47] Olson EA Overbey TA Ostrand CG Pizzagalli DA Rauch SL Rosso IM. Childhood maltreatment experiences are associated with altered diffusion in occipito-temporal white matter pathways. Brain Behav. (2020) 10:e01485. 10.1002/brb3.1485 31773917 PMC6955831

[48] Hardt J Rutter M. Validity of adult retrospective reports of adverse childhood experiences: Review of the evidence. J Child Psychol Psychiatry. (2004) 45:260–73. 10.1111/j.1469-7610.2004.00218.x 14982240

