# Supplementary material for: Adverse Childhood Experience Is Associated With Disrupted White Matter Integrity in Autism Spectrum Disorder: A Diffusion Tensor Imaging Study
Source: Front Psychiatry. 2022 Jan 3;12:823260. doi: 10.3389/fpsyt.2021.823260 (PMC8761790; doi:10.3389/fpsyt.2021.823260)
Supplement: Supplementary file 1 [file Data_Sheet_1.docx]

Supplementary Material

# Supplementary Tables

| Supplementary Table 1. Group differences of demographic characteristics between ASD with low and high CATS. | | | | |
| --- | --- | --- | --- | --- |
|  | ASD with low CATS (n = 31) | ASD with high CATS (n = 32) | T, Umar χ^2^ | *p*-value |
| Age, mean (SD) | 26.9 (6.7) | 27.6 (7.5) | 509 | 0.86 |
| Duration of education, mean (SD) | 15.0 (2.5) | 14.9 (2.1) | 489 | 0.92 |
| IQ, mean (SD) | 103.9 (11.8) | 98.2 (11.7) | 369.5 | 0.082 |
| Sex, male (%) | 23 (74.2) | 25 (78.1) | 0.13 | 0.77 |
| Handedness, right (%) | 29 (93.5) | 29 (90.6) | 0.18 | 1.00 |
| AQ-J, mean (SD) | 30.9 (6.0) | 31.9 (8.5) | 563.5 | 0.35 |
| ADOS-2, mean (SD) | 15.6 (3.4) | 15.3 (2.8) | 492 | 0.96 |
| CATS, mean (SD) |  |  |  |  |
| total | 23.9 (8.6) | 63.5 (21.3) | 992 | < 0.001 |
| punishment | 7.5 (3.7) | 12.7 (4.9) | -4.8 | < 0.001 |
| sexual abuse | 0.2 (0.9) | 0.9 (1.5) | 623 | 0.016 |
| neglect | 8.0 (4.4) | 22.2 (8.2) | -8.6 | < 0.001 |
| emotional abuse | 5.1 (2.6) | 16.8 (6.7) | -9.1 | < 0.001 |
| others | 3.2 (2.2) | 10.9 (6.1) | 896 | < 0.001 |
| ASD, autism spectrum disorder; CATS, Child Abuse Traumatic Scale; IQ, intelligence quotient, AQ-J, Autism Questionnaire. | | | | |
|  | | | | |

| Supplementary Table 2. Group comparisons of each diffusion parameter among ASD with low and high CATS and TD subjects. | | | | | | |
| --- | --- | --- | --- | --- | --- | --- |
|  |  | ASD with low CATS | ASD with high CATS | TD | *F* | *p-value* |
| UF | right FA | 0.413 (0.018) | 0.408 (0.019) | 0.420 (0.022) | 1.6 | 0.22 |
|  | left FA | 0.414 (0.019) | 0.403 (0.020) | 0.417 (0.019) | 2.7 | 0.072 |
|  | right MD | 0.721 (0.033) | 0.719 (0.022) | 0.725 (0.027) | 0.14 | 0.87 |
|  | left MD | 0.741 (0.026) | 0.747 (0.025) | 0.752 (0.023) | 0.47 | 0.63 |
|  | right RD | 0.547 (0.029) | 0.547 (0.018) | 0.546 (0.027) | 0.58 | 0.56 |
|  | left RD | 0.560 (0.024) | 0.569 (0.020) | 0.566 (0.023) | 1.6 | 0.21 |
|  |  |  |  |  |  |  |
| Ci | right FA | 0.516 (0.033) | 0.507 (0.037) | 0.531 (0.029) | 0.78 | 0.46 |
|  | left FA | 0.461 (0.042) | 0.454 (0.045) | 0.483 (0.036) | 2.8 | 0.066 |
|  | right MD | 0.684 (0.035) | 0.686 (0.028) | 0.698 (0.022) | 0.14 | 0.71 |
|  | left MD | 0.673 (0.034) | 0.677 (0.026) | 0.683 (0.026) | 0.50 | 0.48 |
|  | right RD | 0.465(0.030) | 0.469 (0.031) | 0.463 (0.029) | 0.36 | 0.55 |
|  | left RD | 0.486 (0.027) | 0.493 (0.031) | 0.483 (0.030) | 3.0 | 0.052 |
|  |  |  |  |  |  |  |
| ATR | right FA | 0.418 (0.025) | 0.417 (0.026) | 0.423 (0.020) | 0.04 | 0.96 |
|  | left FA | 0.422 (0.020)^†^ | 0.414 (0.016)^†^ | 0.441 (0.020) | 11.9 | < 0.001^*^ |
|  | right MD | 0.688 (0.023) | 0.688 (0.024) | 0.689 (0.018) | 0.61 | 0.54 |
|  | left MD | 0.688 (0.019) | 0.694 (0.021) | 0.696 (0.024) | 1.4 | 0.26 |
|  | right RD | 0.521 (0.022) | 0.521 (0.024) | 0.519 (0.023) | 0.37 | 0.69 |
|  | left RD | 0.516 (0.019)^†^ | 0.526 (0.018)^†, ††^ | 0.514 (0.021) | 6.8 | 0.002^*^ |
| ASD, autism spectrum disorder; TD, typically developed; CATS, Child Abuse and Traumatic Scale; UF, uncinate fasciculus; | | | | | | |
| ATR, anterior thalamic radiation; CST, corticospinal tract; FA, fractional anisotropy; MD, mean diffusivity; RD, radial diffusivity. | | | | | | |
| ^*^; significant group effect of ANCOVA, p<0.083. | | | | | | |
| ^†^; significant difference compared to TD participants, *p*<0.05; ^††^; significant difference compared to ASD with low CATS, *p*<0.05. | | | | | | |
